# Associations between Wage System and Risk Factors for Musculoskeletal Disorders among Construction Workers

**DOI:** 10.1155/2015/513903

**Published:** 2015-10-28

**Authors:** Jeppe Zielinski Nguyen Ajslev, Roger Persson, Lars Louis Andersen

**Affiliations:** ^1^National Research Centre for the Working Environment, Lersø Parkalle 105, 2100 Copenhagen, Denmark; ^2^Department of Psychology, Lund University, 22100 Lund, Sweden; ^3^Physical Activity and Human Performance Group, SMI, Department of Health Science and Technology, Aalborg University, Aalborg, Denmark

## Abstract

Piece rate and performance based wage systems are common in the construction industry. Construction workers are known to have an increased risk of pain and musculoskeletal disorders (MSD). In this cross-sectional questionnaire study, we examined the association between wage system and (1) physical exertion, (2) time pressure, (3) pain, and (4) fatigue. The participants comprised 456 male Danish construction workers working on one of three different wage systems: group based performance wage, individually based performance wage, and time based wage system. The statistical analyses indicated differences between the wage systems in relation to physical exertion (*ηp* = 0.05) and time pressure (*ηp* = 0.03) but not to pain or fatigue. Workers on group based performance wage scored higher (i.e., worse) than workers on individual performance based wage and workers with an hourly/monthly wage. In conclusion, group performance based wage was associated with higher levels of physical exertion and time pressure. Accordingly, group performance based wage can be viewed as a factor that has the potential to complicate prevention of MSD among construction workers. Since performance based wage systems are common in many countries across the world, more attention should be paid to the health effects of these types of payment.

## 1. Introduction

Construction work in Denmark is usually performed by “gangs” of workers within a single profession (e.g., bricklayers, carpenters, and concrete workers). One way to compensate workers within the construction sector is performance based wage. This type of compensation differs from time based/fixed-schedule compensation (e.g., a weekly or monthly wage) in that performance based compensation is dependent on the number of produced units (or some other form of predefined and repetitive work cycle). As such performance based wages may sometimes be called “piece rate wages” and be dependent on individual or group performance. Even if no official documentation regarding the commonness of performance based wages exists, union representatives estimate the percentage to be circa 20–50% of total work.

Even though performance based wages are fairly common in many countries, few studies have examined whether performance based wages are associated with any health consequences [1]. The few extant studies have, however, linked performance based wages with signs of poorer health. For example, in a study from 1989, Vinet et al. reported that the use of stomach medication was higher among piece rate workers than among workers receiving hourly wages [2]. In a more recent study, performance based wages were associated with higher levels of personal and work-related burnout compared with workers receiving wages according to a fixed time based schedule [3]. In a study among Danish slaughterhouse workers, Kristensen documented both higher levels of experienced work strain and higher levels of sickness absence among workers on piece rate wages [4].

Ackroyd and Thompson [5], who studied organizational misbehavior, argued that piece rate wages often resulted in greater effort from workers, an observation that is in agreement with a recent qualitative research study that focused on the work conditions among Danish construction workers [6]. When interviewed, the construction workers identified the piece rate wage as a contributing factor to intensification of work. Piece rate work was in their statements linked to working at higher pace, experiencing time pressure, putting in higher physical effort, and skipping the usage of technical assistive devices. Workers expressed wishes to increase productivity and earnings as a main motivator. Indeed, the ability to work hard and fast to gain economic rewards was central for maintaining a successful professional identity [6].

Increasing the physical effort and work pace in construction work may, however, for several reasons induce health risks. First, physically straining work, characterized by heavy lifting, pulling, or dragging, and work in awkward positions and at high pace are known risk factors for developing musculoskeletal disorders (MSD) in the general working population [7]. Second, the prevalence of MSD is known to be particularly high among workers with manual labor and the risk of long-term sickness absence is higher among blue-collar workers compared to white-collar workers [8]. Likewise, in a recent study among 325,549 Swedish construction workers, heavy physical work was an important predictor for disability pension [9]. According to statistics from Danish working authorities, workers in the construction sector are ranked second only to workers in slaughterhouses regarding prevalence of work-related MSD [10, 11]. In view of these findings, there is reason to suspect that a wage system that urges construction workers to increase the physical effort could induce health risks and be counterproductive to preventing MSD and associated risks (e.g., sickness absence and disability pensioning). 

*Performance Based Wages in the Danish Construction Industry*. The term* piece rate wage* is in Danish construction work today interchangeably assigned to a number of performance based types of payment. For example, the term may refer to package agreements regarding the delivery of a specified product resulting in a specified payment no matter how long time is used. Alternatively, piece rate may refer to payment with a base wage supplemented with a bonus for completing work on time or faster than scheduled. In any event, an inherent quality of performance based wages is that working faster enables workers to earn more money. However, even if the performance based wage system is a monetary agreement between the individual worker, the work gang, and the employer, it is also clear that this method entails social aspects that may affect the work pace and work effort. In fact, an earlier study has shown that social inclusion in the work gang is often dependent on the individual's ability to show physical prowess and to protect and increase the collective earnings of the work gang [6]. In Denmark, piece rate, or performance based, wages may be more or less individually or group based, making the individual worker more or less obliged to consider the collective interests of the gang in terms of maintaining a mutually acceptable level of collective earning. 

*The Present Study*. Against the background outlined above, we decided to examine to what extent performance based wages in the Danish construction industry was associated with construction workers' sense of time pressure and physical discomfort. Because no collected databases on wage systems and physical exposures in Danish construction work currently exist, we decided to conduct a cross-sectional survey study addressing these topics. On basis of previous knowledge, it was expected that working on performance based wages would be associated with higher levels of physical exertion and perceived time pressure. In addition, we expected that fatigue and pain levels would be higher in a performance based wage system, as higher levels of physical exertion were shown to be related to increased musculoskeletal pain [12]. Furthermore, in line with previously mentioned reasons for social inclusion in the work gangs, workers on a* group based* performance wage were expected to experience even higher levels than both workers on* individually based* performance wages and workers on* hourly/monthly based* wages on all of the aforementioned factors. As such, this investigation contributes to our knowledge regarding whether wage systems may pose a barrier for improving workplace health in construction work and also it extends our knowledge regarding the effects of performance based wages on workers' health, particularly risk factors for MSD.

## 2. Methods

### 2.1. Study Design

This is a cross-sectional observational study that was conducted between June and December 2013. Pencil and paper questionnaires were distributed to workers at their workplaces and typically collected a few days later. Participants were eligible for a compensation of up to the worth of 20 euros (in cash or in the form of a gift (i.e., a bottle of wine)). Participation was voluntary and the study was conducted in accordance with Danish Law according to which questionnaire and register-based studies do not need formal ethical approval.

### 2.2. Study Sample

640 construction workers were invited to participate in a questionnaire study. Of these, 519 workers in the ages of 18 to 64 years agreed and responded to the questionnaire (response rate 81%). Three women and 12 persons who provided no information on gender were excluded, leaving 504 male construction workers. Of these, 456 participants gave information on wage system and thus comprised the final study sample. An overview of their demographic characteristics and lifestyle factors is presented in Table 1. Participants were recruited from 81 different construction gangs across Denmark. Four professions were represented: carpentry, bricklaying, scaffolding, and concrete work. These professions were identified as particularly relevant to the MSD challenges in the construction sector [13] and selected in agreement between representatives from the trade union, the employers association, and the researchers. The recruitment of participants was conducted with aid from the Construction Industry's Health and Safety Bus (CIHS-Bus). CIHS-Bus is a small organization with circa 10 employees which assists construction companies, safety representatives, and employees in developing and ensuring safe and healthy working conditions. The service is financed by trade unions and employer associations in the Danish construction industry.

### 2.3. Measures

A tailored questionnaire was compiled. Several items were derived from preceding group and individual interviews and from review literature, whereas other items were derived from extant questionnaires.

#### 2.3.1. Wage System

We assessed the workers predominant means of economic compensation through the question,* “How have you primarily received economic compensation during the last three years?”* The response alternatives were “hourly wages,” “monthly wages,” “individual performance based wages,” and “group performance based wages.” Because hourly and monthly salaries are expressions of time based exchanges of labor for money and the group of workers on monthly wages is relatively small in the construction industry (and in our sample), we grouped these workers into one group (i.e., “hourly/monthly wages”).

#### 2.3.2. Physical Exertion

Participants' degree of perceived physical exertion was measured by the Borg CR10 scale [14, 15]. This scale has previously been validated against physiological measurements during an ordinary work day among blue-collar workers [16] and a modified Borg scale was shown prospectively to increase the risk of sickness absence [17] and musculoskeletal pain [12, 18] in other occupational groups with physically strenuous work. In the present study, participants had to rate how they perceived their average physical exertion in their jobs. The question read “*In general, how physically exerting do you perceive your current work to be?*” The response scale had 16 steps. Higher values indicated greater perceived exertion and the scale steps were 0: not at all, 0.3 and 0.5: extremely weak, 1: very weak, 1.5 and 2: weak, 2.5 and 3: moderate, 4: somewhat strong, 5: strong, 6 and 7: very strong, 8, 9, and 10: extremely strong, and 11: maximal exertion.

#### 2.3.3. Time Pressure

Time pressure was assessed asking, “*How often can you work at a nice and easy pace and still get your work done?*” The question was responded to on a five-point scale: 1: Always, 2: Often, 3: Sometimes, 4: Rarely, and 5: Never. The scores were treated both as a continuous variable (range 1 to 5) and as a trichotomized categorical variable: “Always” and “Often”: 1, “Sometimes”: 2, and “Rarely” and “Never”: 3.

#### 2.3.4. Fatigue

Fatigue was assessed through the question, “*How fatigued are you after a typical work day?*” This item has previously been used in the validation of self-reported physical exertion [19]. The question was responded in relation to six different bodily locations (i) in general, (ii) in the back, (iii) in neck/shoulder, (iv) in arms/wrists, (v) in legs, and (vi) in the head. All items were responded to on a five-step scale indicating the degree of perceived fatigue: 1: not fatigued, 2: a little fatigued, 3: somewhat fatigued, 4: fatigued, and 5: exhausted. Scores from the individual items were used as outcome measure. In addition, a total fatigue score was calculated by averaging the six fatigue items (Cronbach's alpha = 0.85).

#### 2.3.5. Pain

Pain frequency was assessed by asking “*how often do you feel bodily pain (including arms, hands, knees, shoulder, back, etc.)*”. The question was answered on a five-point categorical scale 1: every day, 2: several times a week, 3: a few times a month, 4: a few times a year at maximum, and 5: never. This question was derived from a Danish national study on work health and safety [13].

Regional pain intensity was assessed by asking “on an eleven-point scale ranging from ‘0 (no pain at all)' to ‘10 (worst possible pain)' report the level of pain in (i) neck, (ii) shoulder, (iii) elbows, (iv) hands or wrists, (v) fingers, (vi) lower back, (vii) hips, (viii) knees, (ix) feet or ankles, and (x) in the head.” This question was similar to the one employed by Andersen et al. in a study on physical exercise interventions on physical pain [20]. As with fatigue, a total pain score was calculated by averaging the ten pain items (Cronbach's alpha = 0.85). This score was employed in the adjusted analysis.

### 2.4. Statistical Analysis

The IBM/SPSS 21 software [21] was used and *p* values equal to or below 0.05 were considered statistically significant. Using the General Linear Model (GLM) module in IBM/SPSS, univariate ANOVA *F*-tests were used to examine differences between the three wage conditions (group performance, individual performance, and weekly/monthly wages) and the four continuous outcome scores (physical exertion, fatigue, time pressure, and pain). Because of the few extant studies on wage systems in construction work, we found it relevant to compare the different wage conditions against each other (as opposed to selecting one of them as a reference level). Effect sizes are reported as partial eta square [22]. To evaluate pairwise differences between wage conditions, Bonferroni adjusted post hoc *t*-tests were used for correlated outcomes.

Furthermore, we used Pearson Chi-square test to evaluate associations between the three wage conditions and the categorical variable time pressure (three categories). To evaluate pairwise differences between column proportions, post hoc Bonferroni adjusted *z*-tests were calculated.

To examine whether the reporting of physical exertion, fatigue, pain, and time pressure could be explained by other factors, we used general linear models including age, profession, BMI, smoking habits, leisure time physical activity, vegetable eating habits, and alcohol consummation as control variables. For these multiadjusted analyses, we used the Proc Glm procedure in SAS (version 9.3).

Due to missing replies for some questions, the number of responses in the analysis range from 433 to 456. For questions regarding regional pain intensity, internal missing values range between 366 and 402 as respondents did not register pain in all areas. For adjusted analysis, the total number of analyzed responses ranges from 367 to 383.

## 3. Results

### 3.1. Physical Exertion

Table 2 presents results between wage system and participants perception of the general physical exertion during work. Participants on group performance wages reported an average of 1.18 (95% CI = 0.58 to 1.78) points higher on the Borg scale of physical exertion than participants on hourly/monthly wages and 0.64 (95% CI = −0.16 to 1.44) points higher than on individually performance based wages (*F*[2,436] = 11.45, *p* < 0.001, and *ηp* = 0.05). In the adjusted analysis (Table 4), this association was maintained, differences being 1.13 (95% CI = 0.62 to 1.64) and 0.97 (95% CI 0.30 to 1.64), respectively. Notably, also profession and higher BMI showed significant association to physical exertion.

### 3.2. Time Pressure (Continuous Score)

Table 2 shows that participants on group performance based wages reported 0.36 (95% CI 0.12 to 0.61) points higher on the time pressure scale compared to participants on hourly/monthly wages and 0.28 (95% CI = 0.06 to 0.61) points higher than workers on individually performance based wages (*F*[2,430] = 6.27, *p* = 0.002, and *ηp* = 0.03). In the adjusted analysis (Table 5), this association was maintained, differences being 0.24 (95% CI = −0.06–0.53) and 0.27 (95% CI = 0.05–0.49), respectively.

### 3.3. Time Pressure (Categorical Variable)

Table 3 shows the analysis of time pressure as a categorical variable. This analysis showed that 41.3% of the construction workers on group performance based wages reported “rarely/never” being able to work at a nice and easy pace and still get their work done (*p* = 0.017). In contrast, the corresponding figure among workers on individually performance based wages and hourly/monthly wages was 29.9% and 24.8%, respectively.

### 3.4. Fatigue

In Table 2, it is shown that participants on group performance based wages reported on average 0.27 (95% CI 0.03–0.54) points higher in the hand/wrist region than workers with hourly/monthly wages and 0.13 (95% CI = −0.21 to 0.48) points higher than individually performance based salaries on the five-point scale (*F*[2,438] = 3.8, *p* = 0.023, and *ηp* = 0.02). There were no other statistically significant associations between fatigue and wage system in this analysis. The adjusted analysis of the general fatigue mean showed only association with age.

### 3.5. Pain (Continuous Score)

Pain intensity in the feet/ankle region for participants on individually performance based wages (Table 2) was rated 0.99 (95% CI = 0.15–1.84) points higher than on hourly/monthly salaries and 1.5 (95% CI = 0.51–2.45) points higher than group performance based salaries (*F*[2,376] = 6.86, *p* = 0.001, and *ηp* = 0.04). The adjusted analysis (Table 5) showed that the mean pain intensity was incrementally associated with age.

### 3.6. Pain (Categorical Variable)

When analyzing the single question concerning the frequency of general pain there was no association with wage system (Table 3).

## 4. Discussion

The main finding of this study was that group performance based wages were associated with higher levels of physical exertion and time pressure, while no such association was found for pain and fatigue. However, before discussing the implications and concluding our observations we need to address some methodological issues.

A strength in our study was the fairly large sample size and the fact that we were able to approach construction workers in their working environments, which is likely to reduce any influence of self-selection. However, our choice to assess wage system with a rather crude resolution, focusing on some clear demarcations between wage systems, may be a limitation. This is, however, a limitation that we share with earlier research on wage systems and a limitation that is difficult to resolve [1, 3]. Indeed, the category* performance based wage* encompasses numerous ways of organizing and defining the performance based part. The alternatives range from basis wage with possible bonus for performance to wage systems based entirely on performance. In fact in some negotiated performance based wage systems, workers can potentially work themselves into a deficit, thus earning less than the agreed minimum wage. From this perspective, it is clear that the current abstraction level should be regarded as a feasible compromise between inclusiveness and fragmentation.

Another compromise is the cross-sectional design. Obviously the cross-sectional design limits the possibilities of making trustworthy causal inferences. On the other hand, and due to the conditions of the construction industry (e.g., high turnover of staff and changing workplaces and work gangs), it is very difficult to perform prospective studies with high follow-up rates. Yet, if possible, the prospective perspective should be taken into consideration in future investigations of performance based wage systems. In addition, we have no information whether workers that have a hard time managing, or coping with higher physical exertion, already have shifted away from individual or group based piece work (i.e., the associations may be influenced by the “healthy worker effect”) [23].

A related question is that the assessment of wage system focused on the last 3 years. This time period corresponds to the mean time of employment in our survey (3 years, SD 1.6 years). While this reflects the known high turnover rate in the construction industry, it also hints that some workers have been employed in the current wage system only for a short time. Since a short employment time risk blurring the association between physical exertion and pain, this may lead to more conservative estimates of differences between wage systems. Similarly, that a higher percentage of workers in the group with performance based wages also reported performing high intensity physical activity in their leisure time (Table 1) may similarly reduce the differences between wage groups as physical activity is known to counteract negative effects of heavy physical labor on pain [24, 25].

A limitation is also that all data have been collected with self-reports. While self-reports may contribute useful information on, for example, pain and sickness absence [12, 19], it is also clear that self-reports may be sensitive to extant discourses at work and in society, the respondents ability to recall, beliefs, and many other things [26]. There is also a risk of common method bias [27]. Thus, it cannot be excluded that workers on group based performance wage, who many times appreciate the opportunity to earn some extra money by working fast, perceive their work environment in more positive terms. Possibly, this leads to a more conservative estimate of differences between wage systems. In extension, this raises the question whether the observed differences in physical exertion and pain are practically important. Indeed, the observed magnitude of differences between the three wage systems was generally small to moderate. However, the general pattern of results agrees with results from previous qualitative studies [6]. In addition, the observed differences imply worsening in relation to the average level of physical exertion and time pressure, which in their own right indicate a high workload, irrespective of wage system. Thus, data from different sources seem to signal that construction workers tend to work faster and with less regard to physically exerting work when economic incentives motivate this behavior [6]. As such, our results support the idea that working on group based performance wage may increase time pressure and physical exertion and thereby act counterproductive to preventing MSD in construction work.

To summarize and even if we cannot conclude with any certainty to which extent wage system is a causal factor in the development of pain, which often develops over several years of work, the pattern of results suggests that our classification of wage systems was able to capture some systematic differences. However, it is likely that the difficulties of classification lead to an underestimation of the effects due to the inclusion of residual variance following imperfect measurements.

### 4.1. A Holistic View on Pain and MSD

From a more holistic view on pain and MSD, as viewed through a biopsychosocial perspective, it is known that pain is mediated via more complex mechanisms than mere exposure to physical strain in different forms [28, 29]. For instance, we know from research on the construction industry that there is a high emphasis on self-management within the piece rate gangs, which is appreciated by workers in this type of organization [6, 30, 31]. Theoretically, it is assumed that self-management could lead to increased control over work and therefore help workers to cope with physical exertion and pain as well as the stress from time pressure (cf. [32]). This assumption receives some support from observations made in other occupations, in which a high influence at work has been found to be prospectively associated with less chance of developing back pain [33]. However, self-management may also allow for a regulation of the work within the work gang in a way that increases the workload and the risk of developing pain. To what extent self-management explains the lack of significant association between wage system and perceived musculoskeletal pain in the present study is not known and it is difficult to discern the net effect of self-management on pain. Yet the complexity of the social processes within the work gangs calls for further investigation of the influence on work among workers in piece rate gangs and how influence over work may affect development of MSD.

That age was the only significant factor that was associated with pain intensity in the adjusted analyses deserves a brief comment. In MSD research, there is a healthy debate on whether certain jobs lead to increased risk of developing MSD or whether these diseases should mainly be ascribed to effects of “normal” human ageing [34, 35]. From this perspective, it may be noted that a national survey encompassing the general Danish working population, found that men in the ages 25–49 on average experienced a 20% higher average pain intensity than men between 18 and 24 years (on a mean score calculated from ratings of pain in the lower back, hand/elbow, and shoulder/neck regions [36]). In our study, the average difference between age categories in the same body regions was 34%. Similar increases were observed for workers above 50 years. As such, the difference between the youngest and eldest groups was 65% in our study of construction workers compared with 44% in the general working population. This observation of differences seems to support the notion that construction work is associated with higher long-term development of MSD. Studies on effective prevention of MSD in this high-risk occupation are needed.

## 5. Conclusion

The study shows that working on group performance based wages is associated with reporting higher levels of physical exertion and time pressure, compared with working on hourly/monthly wages. Group based performance wage may contribute as a barrier to preventing MSD by altering workers' orientation towards working faster and with less regard to their bodily wellbeing.

## Figures and Tables

**Table 1:** Background information.

	Total data	Hourly/monthly paid	Individual performance based wages	Group performance based wages
Number of participants	456	273	72	111
Profession (*N*)				
Bricklayer's labourers^*β*^	23	11	5	7
Bricklayer	81	38	11	22
Concrete workers	136	85	17	34
Scaffolders	57	24	14	19
Carpenters	143	95	23	25
Age (mean years (SD))	40 (12.6)	39 (12.9)	45 (10.8)	40 (12.2)
Height (mean cm (SD))	181 (6.8)	181 (6.9)	180 (6.6)	181 (6.97)
Weight (mean kg (SD))	85 (13.0)	86 (13.7)	87 (11.8)	85 (12.1)
BMI (mean kg/m^2^ (SD))	26.2 (3.7)	26.2 (3.9)	26.7 (3.6)	25.9 (3.4)
Smokers (%)	38	39	31	41
Sleep deprived^*∗*^ (%)	8	8	9	10
Leisure time fitness activity				
Medium intensity physical activity				
More than 2 hours/week (%)	48	49	37	53
Less than 2 hours/week (%)	52	51	63	47
High intensity physical activity (HIPA)				
Performs HIPA (%)	40	39	32	47
Never performs HIPA (%)	60	61	68	53
Vegetable eating habits				
Daily (%)	36	38	32	33
Less than daily (%)	64	62	68	67
Units of alcohol per day (Monday to Friday)				
Maximum 2 units/day (%)	83	86	80	76
More than 2 units/day (%)	17	14	20	24

Note: SD, standard deviation.

^*∗*^Defined as the percentage registering rarely getting sufficient sleep.

^*β*^Bricklayer's labourers in Danish construction work are workers who make sure bricklayers have all the materials they need and that the work site is ready to work and is clean and tidy and also plan work ahead. This is a very traditional organization of bricklaying.

**Table 2:** Unadjusted mean scores across wage system groups.

	Total		Hourly/monthly paid (H)		Individual performance wage (I)		Group performance wage (G)		Univariate ANOVA *F*-test	
	Mean	SD	Mean	SD	Mean	SD	Mean	SD	*p* value	Partial eta square
Physical exertion (Borg CR-10)	5.82	(2.2)	5.45	(2.2)	5.99	(2.3)	6.63^*∗∗*^	(2.0)	<0.001	0.05
Time pressure	3.10	(0.9)	3.00	(0.9)	3.09	(1.0)	3.37^*∗∗*^	(0.9)	0.002	0.03
Fatigue										
Total score	2.79	(0.7)	2.73	(0.7)	2.88	(0.7)	2.87	(0.7)	0.105	—
General	3.01	(0.8)	3.00	(0.8)	2.96	(0.8)	3.07	(0.8)	0.581	—
Back	2.92	(0.9)	2.87	(1.0)	3.06	(0.9)	2.94	(0.9)	0.291	—
Neck/shoulder	2.80	(1.0)	2.72	(1.0)	2.94	(0.9)	2.89	(0.9)	0.117	—
Arms/wrist	2.67	(0.9)	2.58	(0.9)	2.73	(1.0)	2.86^*∗∗*^	(0.9)	0.023	0.02
Legs	2.83	(0.9)	2.78	(1.0)	2.87	(1.0)	2.93	(0.9)	0.357	—
Head (mental)	2.51	(0.9)	2.47	(0.9)	2.69	(1.0)	2.49	(0.8)	0.191	—
Pain										
Neck	3.04	(2.3)	2.95	(2.4)	3.2	(2.3)	3.18	(2.2)	0.605	—
Shoulder	3.84	(2.6)	3.66	(2.6)	4.13	(2.4)	4.10	(2.6)	0.244	—
Elbows	2.61	(2.2)	2.42	(2.2)	2.77	(2.1)	2.98	(2.3)	0.114	—
Hands/wrists	3.22	(2.3)	3.09	(2.3)	3.12	(2.0)	3.57	(2.4)	0.219	—
Fingers	2.88	(2.3)	2.92	(2.4)	2.89	(2.1)	2.77	(2.2)	0.858	—
Lower back	4.16	(2.9)	3.99	(2.9)	4.67	(2.9)	4.24	(2.8)	0.235	—
Hips	2.63	(2.3)	2.63	(2.5)	3.12	(2.1)	2.34	(2.0)	0.132	—
Knees	4.27	(2.8)	4.25	(2.9)	4.13	(2.3)	4.4	(2.9)	0.841	—
Feet/ankles	2.93	(2.4)	2.89	(2.5)	3.88^†^	(2.6)	2.4^*∗*^	(1.9)	0.001	0.04
Head	2.19	(1.9)	2.17	(1.8)	2.48	(2.3)	2.04	(1.7)	0.362	—

Note: ^*∗*^post hoc Bonferroni adjusted *p* value (*p* < 0.05) between G and I.

^*∗∗*^Post hoc Bonferroni adjusted *p* value (*p* < 0.05) between G and H.

^†^Post hoc Bonferroni adjusted *p* value (*p* < 0.05) between I and H.

Borg-CR-10 scores range from 0 to 11: 0: not all exerted, 10: extremely strong, and 11: maximal exertion. Time pressure score range between 1 and 5: 1: always and 5: never in relation to the question: *How often can you work at an nice and easy pace and still get your work done?* Fatigue scores range from 1 to 5: 1: not fatigued and 5: exhausted. Pain scores range from 1 to 5: 1: every day and 5: never.

**Table 3:** Categorical analysis for wage system on time pressure and general pain.

	Total		Hourly/monthly paid (H)		Individual performance wage (I)		Group performance wage (G)		Chi-square test
	*N*	%	*N*	%	*N*	%	*N*	%	*p*-value
Time pressure^*β*^									
Always/Often	(98)	22.6	(65)	24.8^*∗∗*^	(18)	26.9^*∗*^	(15)	14.4	
Sometimes	(207)	47.8	(132)	50.4	(29)	44.2	(46)	44.2	
Rarely/Never	(128)	29.6	(65)	24.8	(20)	29.9	(43)	41.3^*∗∗*^	
									0.017
Pain general									
Every day	(120)	27.1	(68)	25.7	(19)	27.5	(33)	30.6	
Several times a week	(126)	28.5	(75)	28.3	(20)	29	(31)	28.7	
A few times a month	(127)	28.7	(78)	29.4	(20)	29	(29)	26.9	
A few times a year	(59)	13.3	(35)	13.2	(9)	13	(15)	13.9	
Never	(10)	2.3	(9)	3.4	(1)	1.4	(0)	0	
									0.182

Note: Between-condition comparisons for categorical data were made with Pearson's Chi-squared test.

^*β*^Note: Time pressure was assessed by the question: “*How often can you work at an nice and easy pace and still get your work done?”*.

^*∗*^Pair-wise difference between G and I, *p* < 0.05.

^*∗∗*^Pair-wise difference between G and H, *p* < 0.05.

**Table 4:** Physical exertion and fatigue scores adjusted for individual, demographical, and lifestyle factors.

	Physical exertion (Borg CR-10) (*n* = 373)			Fatigue (*n* = 383)		
	Mean	95% CI	*p* value	Mean	95% CI	*p* value
Age			0.353			0.003
(1) 18–29	6.34	5.83–6.85		2.69^*µβ*^	2.53–2.86	
(2) 30–39	6.47	5.96–6.98		2.83^*µβ*^	2.67–2.99	
(3) 40–49	6.71	6.23–7.19		3.05^*∗*†^	2.89–3.20	
(4) 50–59	6.36	5.85–6.86		3.04^*∗*†^	2.88–3.21	
(5) 60+	5.58	4.54–6.62		2.79^*µβ*^	2.45–3.13	
Profession			0.001			0.053
(1) Bricklayer labourer	7.13^†*µ*#^	6.18–8.08		2.93	2.62–3.24	
(2) Bricklayer	5.96^*∗β*^	5.41–6.51		2.82	2.64–3.00	
(3) Concrete worker	6.06^*∗β*#^	5.6–6.53		3.03	2.88–3.18	
(4) Scaffolder	6.79^†*µ*#^	6.21–7.37		2.85	2.67–3.04	
(5) Carpenter	5.51^*∗µβ*^	5.03–5.99		2.77	2.61–2.92	
BMI			0.044			0.423
(1) Normal weight	6.13^*µ*^	5.71–6.54		2.88	2.74–3.01	
(2) Overweight	5.93^*µ*^	5.54–6.36		2.82	2.68–2.95	
(3) Obese	6.80^*∗*†^	6.18–7.42		2.95	2.75–3.15	
Smoking			0.695			0.142
(1) No	6.24	5.86–6.63		2.83	2.70–2.95	
(2) Yes	6.34	5.89–6.78		2.94	2.79–3.08	
Physical activity			0.550			0.810
(1) No	6.22	5.82–6.62		2.89	2.76–3.02	
(2) Yes	6.36	5.93–6.79		2.87	2.73–3.01	
Vegetable eating habits			0.092			0.281
(1) 1-2 times a week at most	6.49	6.05–6.93		2.92	2.78–3.07	
(2) 3–6 times a week at least	6.09	5.71–6.48		2.84	2.72–2.97	
Intake of alcohol			0.088			0.982
(1) ≤1 unit a day	6.51	6.1–6.92		2.88	2.75–3.01	
(2) >1 unit a day	6.07	5.63–6.51		2.88	2.74–3.02	
Wage system		—	<0.001			0.105
(1) Group performance wage	6.99^†*µ*^	6.51–7.47		2.98	2.83–3.14	
(2) Individual performance wage	6.02^*∗*^	5.45–6.58		2.86	2.68–3.05	
(3) Hourly/monthly wage	5.86^*∗*^	5.46–6.27		2.80	2.67–2.93	

Note: ^*∗*^is post hoc *t*-test, pairwise difference from level 1, *p* < 0.05, ^†^is post hoc *t*-test, pairwise difference from level 2, *p* < 0.05, ^*µ*^is post hoc *t*-test, pairwise difference from level 3, *p* < 0.05,  ^*β*^is post hoc *t*-test, pairwise difference from level 4, *p* < 0.05, and ^#^is post hoc *t*-test, pairwise difference from level 5, *p* < 0.05.

**Table 5:** Time pressure and pain intensity scores, adjusted for individual, demographical, and lifestyle factors.

	Time pressure (continuous) (*n* = 375)			Pain (*n* = 367)		
	Mean	95% CI	*p* value	Mean	95% CI	*p* value
Age			0.76			0.003
(1) 18–29	3.17	2.95–3.39		2.29^*µβ*#^	1.81–2.77	
(2) 30–39	3.32	3.10–3.54		2.64^*β*#^	2.16–3.12	
(3) 40–49	3.26	3.05–3.48		3.04^*∗*^	2.58–3.49	
(4) 50–59	3.20	2.98–3.42		3.33^*∗*†^	2.84–3.82	
(5) 60+	3.11	2.64–3.58		3.89^*∗*†^	2.91–4.87	
Profession			0.03			0.127
(1) Bricklayer labourer	3.60	3.19–4.02		3.45	2.53–4.38	
(2) Bricklayer	3.11	2.87–3.35		2.85	2.34–3.37	
(3) Concrete worker	3.24	3.03–3.44		3.32	2.88–3.75	
(4) Scaffolder	3.04	2.79–3.30		2.89	2.34–3.45	
(5) Carpenter	3.07	2.86–3.28		2.67	2.22–3.13	
BMI			0.35			0.119
(1) Normal weight	3.24	3.06–3.42		3.13	2.74–3.52	
(2) Overweight	3.13	2.95–3.31		2.73	2.35–3.12	
(3) Obese	3.27	2.99–3.54		3.25	2.66–3.84	
Smoking			0.92			0.488
(1) No	3.22	3.05–3.39		2.96	2.60–3.33	
(2) Yes	3.20	3.01–3.40		3.12	2.70–3.53	
Physical activity			0.45			0.445
(1) No	3.20	3.02–3.38		3.12	2.74–3.51	
(2) Yes	3.23	3.04–3.41		2.95	2.55–3.36	
Vegetable eating habits			0.24			0.521
(1) 1-2 times a week at most	3.15	2.96–3.35		3.11	2.69–3.53	
(2) 3–6 times a week at least	3.27	3.10–3.44		2.97	2.60–3.33	
Intake of alcohol			0.06			0.196
(1) ≤1 unit a day	3.13	2.95–3.31		3.19	2.80–3.58	
(2) >1 unit a day	3.30	3.11–3.49		2.89	2.47–3.30	
Wage system		—	0.05			0.583
(1) Group performance wage	3.38^*µ*^	3.17–3.60		3.14	2.69–3.59	
(2) Individual performance wage	3.15	2.89–3.40		3.08	2.54–3.62	
(3) Hourly/monthly wage	3.11	2.93–3.29		2.90	2.52–3.28	

Note: ^*∗*^is post hoc *t*-test, pairwise difference from level 1, *p* < 0.05, ^†^is post hoc *t*-test, pairwise difference from level 2, *p* < 0.05,  ^*µ*^is post hoc *t*-test, pairwise difference from level 3, *p* < 0.05,  ^*β*^is post hoc *t*-test, pairwise difference from level 4, *p* < 0.05, and ^#^is post hoc *t*-test, pairwise difference from level 5, *p* < 0.05.
